# A Coupled Inflammation–Metabolism Index for Early Prediction of Organ Dysfunction Progression Within 24 h After ICU Admission in Septic Shock

**DOI:** 10.1155/emmi/1308518

**Published:** 2026-09-09

**Authors:** Weiying Ding, Qiang Shen, Yingjie Geng

**Affiliations:** ^1^ Department of Critical Care Medicine, Beijing Shijingshan Hospital, Shijingshan Teaching Hospital of Capital Medical University, Beijing 100043, China

**Keywords:** IL-6, inflammation–metabolism coupling, organ dysfunction progression, septic shock

## Abstract

**Background:**

Early progression of organ dysfunction is common in septic shock and is closely associated with poor outcomes. Reliable tools that integrate key pathophysiological processes for early risk stratification remain limited. This study aimed to develop and internally validate a coupled inflammation–metabolism coupling index (IMCI) for early prediction of organ dysfunction progression within 24 h after intensive care unit (ICU) admission in patients with septic shock.

**Methods:**

In this single‐center retrospective observational study, 210 adult patients with septic shock admitted to the ICU between January 2020 and June 2025 were included. Organ dysfunction progression was defined as an increase in Sequential Organ Failure Assessment (SOFA) score ≥ 2 within 24 h. Baseline demographic characteristics, laboratory indicators, disease severity scores, and treatment‐related variables, including white blood cell count, platelet count, Acute Physiology and Chronic Health Evaluation II (APACHE II) score, vasopressor dose, continuous renal replacement therapy, mechanical ventilation, and glucocorticoid administration, were collected and compared between groups. IMCI‐A was constructed as ln(IL‐6) × ln(lactate), and an extended index (IMCI‐B) was evaluated in sensitivity analyses. Logistic regression, receiver operating characteristic analysis, calibration curves, bootstrap validation, and decision curve analysis were performed to assess association, predictive performance, and clinical utility.

**Results:**

Organ dysfunction progression occurred in 88 patients (41.9%). Patients with organ dysfunction progression had a higher inflammatory and metabolic burden and more severe baseline illness than those without progression. In the fully adjusted model incorporating demographic variables, baseline SOFA score, laboratory indicators, disease severity, and treatment‐related variables, IMCI‐A remained independently associated with 24‐h organ dysfunction progression (adjusted OR = 1.68, 95% CI: 1.18–2.39; *p* = 0.004). IMCI‐A demonstrated excellent discriminative performance (area under the curve [AUC] = 0.929), which further improved when combined with SOFA (AUC = 0.948). Calibration and decision curve analyses showed good agreement and favorable net clinical benefit.

**Conclusions:**

IMCI provides a robust and clinically meaningful tool for early prediction of organ dysfunction progression in septic shock, offering incremental value beyond conventional organ function assessment.

## 1. Introduction

Septic shock remains a leading cause of mortality and resource utilization in intensive care units (ICUs) worldwide, despite advances in antimicrobial therapy and organ support strategies [1, 2]. Characterized by profound circulatory, cellular, and metabolic abnormalities, septic shock is associated with a high risk of rapid organ dysfunction progression, particularly during the early phase after ICU admission. Timely identification of patients at risk for early organ deterioration is therefore crucial for optimizing monitoring intensity, guiding therapeutic escalation, and improving clinical outcomes.

The pathophysiology of septic shock is driven by a complex interplay between dysregulated host inflammation and metabolic derangements [3]. Excessive activation of inflammatory pathways, reflected by elevated cytokines such as interleukin‐6 (IL‐6), contributes to endothelial injury, microcirculatory dysfunction, and impaired oxygen utilization [4]. Concurrently, metabolic stress and tissue hypoperfusion, commonly manifested as hyperlactatemia, indicate an imbalance between oxygen delivery and cellular energy demand [5, 6]. Importantly, accumulating evidence suggests that inflammatory burden and metabolic dysfunction do not operate in isolation but rather interact synergistically to accelerate organ injury [7, 8]. However, most existing prognostic tools or biomarkers evaluate these domains separately, potentially underestimating their combined impact on early organ dysfunction.

The Sequential Organ Failure Assessment (SOFA) score is widely used to quantify organ dysfunction severity in sepsis and septic shock [9]. While baseline SOFA provides valuable prognostic information, it primarily reflects established organ injury and may be less sensitive to dynamic pathophysiological interactions occurring during the earliest stages of critical illness [10]. Single inflammatory or metabolic markers, although informative, often show limited discriminative power when used alone [11]. There remains a clear unmet need for an integrated, easily obtainable index that captures the coupling between inflammation and metabolic stress to improve early risk stratification.

In this context, we proposed a coupled inflammation–metabolism coupling index (IMCI) that integrates inflammatory intensity and metabolic disturbance into a unified quantitative metric. We hypothesized that IMCI would be independently associated with early organ dysfunction progression and would provide incremental predictive value beyond conventional clinical scores. Therefore, this study aimed to examine the association between IMCI and organ dysfunction progression within 24 h after ICU admission in patients with septic shock and evaluate its predictive performance, calibration, and potential clinical utility.

## 2. Materials and Methods

### 2.1. Study Design and Subjects

This study was a single‐center, retrospective observational study that included 210 patients with septic shock who were admitted to the ICU between January 2020 and June 2025. The study protocol was approved by the institutional ethics committee and conducted in accordance with the principles of the Declaration of Helsinki. Owing to the retrospective nature of the study, the requirement for informed consent was waived.

### 2.2. Sample Size Estimation

The sample size was estimated based on the requirement to evaluate the predictive performance of inflammation‐ and metabolism‐related indicators for organ dysfunction progression within 24 h. A ΔSOFA score ≥ 2 was defined as the primary outcome. According to previous studies, the area under the receiver operating characteristic (ROC) curve (area under the curve [AUC]) for single inflammatory or metabolic markers in patients with sepsis generally ranges from 0.70 to 0.80 [12]. We therefore hypothesized that combining inflammatory and metabolic information would yield improved discriminatory performance.

Assuming a two‐sided *α* of 0.05 and a statistical power (1‐β) of 0.80, the required sample size for detecting a statistically significant difference between the AUC and the null value of 0.5 was estimated using the method described by Hanley and McNeil for ROC analysis. Given an expected event rate of approximately 40% in the study population, the minimum required sample size was calculated to be about 180 patients. To account for potential missing data and to ensure the robustness of statistical analyses, a total of 210 patients were ultimately included.

### 2.3. Inclusion and Exclusion Criteria

The inclusion criteria were as follows: diagnosis of septic shock according to the Sepsis‐3 criteria [13], defined as (1) suspected or confirmed infection accompanied by persistent hypotension requiring vasopressor therapy to maintain a mean arterial pressure ≥ 65 mmHg and a serum lactate level > 2 mmol/L; (2) age ≥ 18 years, and (3) completion of inflammatory marker assessment, blood gas analysis, and organ function scoring within 24 h after ICU admission.

The exclusion criteria were as follows: (1) ICU length of stay < 24 h; (2) absence of key clinical data (IL‐6, lactate, or SOFA score); and (3) the presence of advanced malignancy or other end‐stage diseases. Patients with missing key variables were excluded from the complete case analysis. Because an ICU stay of < 24 h or an incomplete 24‐h SOFA assessment could have resulted from early death, transfer, or other rapid clinical transitions, the possibility of clinically driven missingness could not be excluded.

### 2.4. Data Collection and Variable Definitions

Demographic characteristics, laboratory results, and organ function scores were retrospectively extracted from the hospital electronic medical record system. Demographic data included age and sex at ICU admission. Underlying comorbidities, including hypertension, diabetes mellitus, chronic kidney disease, and chronic cardiovascular disease, were also collected for baseline comparison across IMCI‐A tertiles. Laboratory indicators included IL‐6, high‐sensitivity C‐reactive protein (hsCRP), arterial lactate, white blood cell (WBC) count, and platelet (PLT) count. Laboratory predictors used for IMCI construction and regression analyses were obtained from the first available measurements after ICU admission and before the assessment of the 24‐h SOFA score in order to reflect the early inflammatory burden, metabolic stress, hematological status, and coagulation‐related changes of the patients.

Inflammation‐related indicators included IL‐6 and hsCRP. IL‐6 was measured using enzyme‐linked immunosorbent assay (ELISA), with results expressed in pg/mL. hsCRP was measured by immunoturbidimetric assay and reported in mg/L. Metabolic/perfusion status was assessed using arterial blood lactate levels (mmol/L) obtained from concomitant arterial blood gas analysis and measured by a bedside blood gas analyzer.

Organ function was evaluated using the SOFA score. Baseline SOFA was defined as the first SOFA score recorded at ICU admission, while the 24‐h SOFA score was defined as the value closest to 24 h after ICU admission. The acute physiology and chronic health evaluation (APACHE II) score was calculated based on clinical and laboratory data obtained within the first 24 h after ICU admission. Treatment‐related variables were also collected to evaluate baseline treatment intensity and potential confounding. These variables included vasopressor dose, use of continuous renal replacement therapy (CRRT), mechanical ventilation, and glucocorticoid administration within the first 24 h after ICU admission. The vasopressor dose was recorded as the norepinephrine‐equivalent dose when multiple vasoactive agents were used. CRRT, mechanical ventilation, and glucocorticoid administration were recorded as binary variables.

All SOFA components were calculated according to standard definitions, encompassing six organ systems: respiratory, coagulation, hepatic, cardiovascular, neurological, and renal systems. All raw variables were examined for completeness prior to statistical analysis. Because IL‐6 and hsCRP exhibited skewed distributions, natural logarithmic transformation was applied during regression analyses and index construction to minimize the influence of extreme values on the results.

### 2.5. Outcome definition

The primary outcome of this study was organ dysfunction progression within 24 h after ICU admission, defined as an increase in the SOFA score of ≥ 2 points from baseline (ΔSOFA ≥ 2). Based on this criterion, patients were classified into a deterioration group and a non‐deterioration group for subsequent comparative analyses and evaluation of predictive performance.

### 2.6. Construction of IMCI

IMCI was proposed and constructed to quantify the combined risk associated with inflammatory intensity and metabolic dysregulation. In the primary analysis, IMCI‐A was defined as IMCI‐A = ln(IL‐6) × ln(lactate). IL‐6 and lactate were natural log‐transformed prior to calculation to reduce skewness and improve index stability. To compare the multiplicative formulation with a simple additive approach, an additive model was also constructed as IMCI‐additive = Z[ln(IL‐6)] + Z[ln(lactate)], where Z denotes standardization. The predictive performance of IMCI‐A and IMCI‐additive was subsequently compared. To further examine baseline differences and outcome trends across different IMCI‐A levels, patients were stratified into low, intermediate, and high IMCI‐A groups according to tertiles of IMCI‐A. Baseline characteristics were then compared across these tertile groups. In sensitivity analyses, an extended coupling index was constructed as IMCI‐B = [Z(ln(IL‐6)) + Z(ln(hsCRP))] × (ln(lactate)) to incorporate a broader inflammatory burden and assess the robustness of the findings.

### 2.7. Regression Analysis and Sensitivity Analysis

ΔSOFA ≥ 2 defined as the outcome, univariable logistic regression analyses were first performed to evaluate the associations of demographic characteristics, laboratory indicators, disease severity scores, treatment‐related variables, and IMCI with 24‐h organ dysfunction progression. Variables of clinical relevance and variables showing statistical significance in univariable analyses were considered for multivariable logistic regression analyses. In the primary multivariable model, IMCI‐A was adjusted for age, sex, and baseline SOFA score. To further address potential residual confounding, sensitivity analyses were performed using extended adjustment models incorporating clinically meaningful potential confounders. Model 1 was unadjusted. Model 2 was adjusted for age, sex, and baseline SOFA score. Model 3 was further adjusted for WBC count, PLT count, and APACHE II score. Model 4 additionally included treatment‐related variables, including vasopressor dose, CRRT, mechanical ventilation, and glucocorticoid administration within the first 24 h after ICU admission. In these sensitivity analyses, WBC count, PLT count, APACHE II score, and vasopressor dose were analyzed as continuous variables, whereas CRRT, mechanical ventilation, and glucocorticoid administration were analyzed as categorical variables. To assess potential multicollinearity between IMCI‐A and baseline SOFA score in the combined model, the variance inflation factor (VIF) was calculated. A VIF value < 5 was considered to indicate no severe multicollinearity. The same regression strategy was also applied to the extended coupling index IMCI‐B to evaluate the robustness of the findings. To account for potential mathematical coupling and ceiling effect related to baseline SOFA score, we performed sensitivity analyses stratified by baseline SOFA tertiles and repeated the analysis after excluding patients in the highest baseline SOFA quartile. The models were adjusted for age, sex, WBC count, PLT count, APACHE II score, vasopressor dose, CRRT, mechanical ventilation, and glucocorticoid administration.

### 2.8. ROC Curve Analysis and Internal Validation

ROC curve analyses were performed to evaluate the discriminative ability of IL‐6, lactate, baseline SOFA score, IMCI‐A, and the combined SOFA + IMCI‐A model for predicting organ dysfunction progression within 24 h. The AUC was calculated for each model. The optimal cutoff value was determined using the Youden index, based on which sensitivity, specificity, accuracy, positive predictive value, and negative predictive value were calculated. To assess the robustness of predictive performance and the potential risk of overfitting, internal validation was conducted using a bootstrap resampling approach with 1000 repetitions. The mean AUC and its 95% confidence interval (CI) were estimated to evaluate the stability of the discriminative ability of the indices.

### 2.9. Calibration Curve Analysis

To assess the agreement between IMCI‐predicted probabilities and the observed incidence of organ dysfunction progression within 24 h, calibration curves were constructed, with the 45° line serving as the reference for perfect calibration. The Brier score was also calculated to quantify overall prediction error. Calibration performance was evaluated comprehensively based on the shape of the calibration curves and the Brier score.

### 2.10. Decision Curve Analysis

Decision curve analysis was performed to evaluate the clinical net benefit of IMCI across a range of threshold probabilities. By comparing the net benefit of IMCI with the strategies of “treat all” and “treat none”, the potential clinical utility of IMCI in risk stratification and decision‐making was assessed.

### 2.11. Statistical Analysis

All statistical analyses were conducted using SPSS Version 26.0 and Python Version 3.11. Continuous variables were tested for normality using the Shapiro–Wilk test. Normally distributed variables are presented as mean ± standard deviation and were compared between groups using the independent‐samples *t* test for two‐group comparisons or one‐way analysis of variance for comparisons across IMCI‐A tertiles. Non‐normally distributed variables are presented as median (interquartile range) and were compared using the Mann–Whitney U test or Kruskal–Wallis test, as appropriate. Categorical variables are expressed as counts and percentages and were compared using the *χ* ^2^ test or Fisher’s exact test, as appropriate. Differences between AUCs were compared using the DeLong test. All tests were two‐sided, and a *p* value < 0.05 was considered statistically significant.

## 3. Results

### 3.1. Baseline Characteristics

**FIGURE 1:**
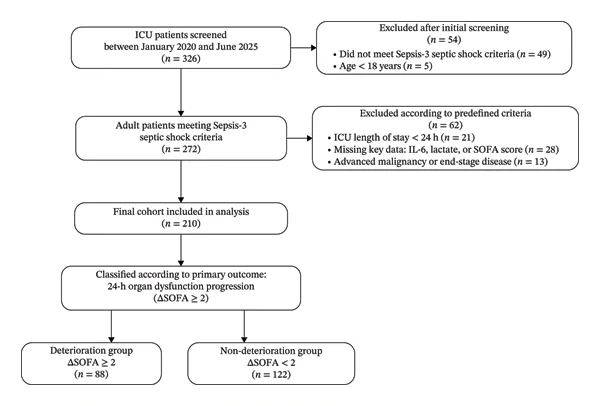
Patient screening flow diagram.

The patient screening and selection process are shown in Figure 1. A total of 210 patients with septic shock were included in this study, of whom 88 (41.90%) developed organ dysfunction progression within 24 h after ICU admission (ΔSOFA ≥ 2). There were no statistically significant differences between the deterioration and nondeterioration groups with respect to age or sex distribution. Compared with the nondeterioration group, patients in the deterioration group had significantly higher WBC counts and lower PLT counts (both *p* < 0.05), indicating more pronounced inflammatory activation and coagulation‐related abnormalities. Inflammatory and metabolic indicators were also markedly increased in the deterioration group. Specifically, IL‐6, hsCRP, and lactate levels were significantly higher in patients with organ dysfunction progression than in those without deterioration (all *p* < 0.05). In addition, the deterioration group showed significantly higher baseline SOFA scores, APACHE II scores, and SOFA scores at 24 h after ICU admission (all *p* < 0.001), suggesting more severe initial illness and subsequent progression of organ dysfunction. Regarding treatment‐related variables within the first 24 h after ICU admission, patients in the deterioration group required higher vasopressor doses and were more likely to receive CRRT, mechanical ventilation, and glucocorticoid therapy than those in the nondeterioration group (all *p* < 0.05). These findings indicate that patients with early organ dysfunction progression had greater inflammatory and metabolic burden, more severe baseline disease status, and higher treatment intensity during the early ICU period (Table 1).

**TABLE 1:** Baseline characteristics stratified by outcome.

Variable	Deterioration group (*n* = 88)	Nondeterioration group (*n* = 122)	Statistical value	*p* value
Age (year), mean ± SD	66.30 ± 12.10	64.80 ± 13.20	*t* = 0.85	0.401
Male, *n* (%)	60 (68.18)	78 (63.93)	*χ* ^2^ = 0.24	0.622
WBC count, × 10^9^/L, median (IQR)	15.20 (10.80–21.30)	12.40 (8.60–17.80)	*Z* = −2.38	0.017
PLT count, × 10^9^/L, median (IQR)	92.00 (58.00–146.00)	128.00 (82.00–191.00)	*Z* = −2.92	0.004
IL‐6 (pg/mL), median (IQR)	260.00 (150.00–520.00)	140.00 (80.00–250.00)	*Z* = −5.12	< 0.001
hsCRP (mg/L), median (IQR)	115.00 (70.00–180.00)	85.00 (50.00–130.00)	*Z* = −3.45	0.001
Lactate (mmol/L), mean ± SD	3.68 ± 1.55	2.74 ± 1.20	*t* = 4.75	< 0.001
Baseline SOFA score, mean ± SD	10.45 ± 3.10	8.85 ± 3.20	*t* = 3.64	< 0.001
APACHE II score, mean ± SD	25.80 ± 6.20	21.60 ± 5.70	*t* = 5.01	< 0.001
Vasopressor dose, μg/kg/min, median (IQR)	0.36 (0.22–0.62)	0.22 (0.12–0.40)	*Z* = −4.01	< 0.001
CRRT, *n* (%)	30 (34.09)	20 (16.39)	*χ* ^2^ = 7.88	0.005
Mechanical ventilation, *n* (%)	64 (72.73)	62 (50.82)	*χ* ^2^ = 9.33	0.002
Glucocorticoid administration, *n* (%)	41 (46.59)	39 (31.97)	*χ* ^2^ = 4.04	0.045
SOFA score at 24 h, mean ± SD	12.78 ± 3.40	8.38 ± 3.10	*t* = 9.60	< 0.001

*Note:* PLT: platelet; IL‐6: interleukin‐6; hsCRP: high‐sensitivity C‐reactive protein; APACHE II: Acute Physiology and Chronic Health Evaluation II; vasopressor dose was expressed as a norepinephrine‐equivalent dose.

Abbreviations: CRRT, continuous renal replacement therapy; SOFA, sequential organ failure assessment; WBC, white blood cell.

### 3.2. Univariable Analysis of Inflammatory and Metabolic Indicators Associated With 24‐h Organ Dysfunction Progression

To further evaluate the predictive value of inflammation‐ and metabolism‐related indicators measured within 24 h after ICU admission for early organ dysfunction progression, univariable logistic regression analyses were performed with ΔSOFA ≥ 2 as the outcome. The results showed that IL‐6, hsCRP, lactate, and baseline SOFA score were all significantly associated with organ dysfunction progression within 24 h (all *p* < 0.05). Specifically, higher levels of inflammatory markers were associated with an increased risk of deterioration, and elevated lactate levels, reflecting metabolic stress and impaired perfusion, were also significantly associated with a higher risk of deterioration. In addition, each 1‐point increase in baseline SOFA score was associated with a corresponding increase in the risk of organ dysfunction progression. Age and sex were not significantly associated with the outcome in the univariable analyses (*p* > 0.05) (Table 2).

**TABLE 2:** Univariable logistic regression analysis for 24‐h organ dysfunction progression.

Variable (unit/scale)	OR	95% CI	Statistical value	*p* value
Age (per 1‐year increase)	1.01	0.99–1.03	*Z* = 0.86	0.390
Male (yes vs. no)	1.21	0.70–2.10	*χ* ^2^ = 0.46	0.498
IL‐6 (ln‐transformed, per 1‐unit increase)	1.78	1.42–2.23	*Z* = 5.11	< 0.001
hsCRP (ln‐transformed, per 1‐unit increase)	1.39	1.10–1.77	*Z* = 2.74	0.006
Lactate (per 1 mmol/L increase)	1.52	1.25–1.85	*Z* = 4.21	< 0.001
Baseline SOFA score (per 1‐point increase)	1.17	1.08–1.27	*Z* = 3.90	< 0.001

*Note:* IL‐6 and hsCRP were natural logarithm‐transformed (ln) prior to inclusion in the regression model.

### 3.3. Multivariable Logistic Regression Analysis: Identification of Independent Predictors

Based on the univariable analyses, IL‐6, hsCRP, lactate, baseline SOFA score, and the clinically relevant covariates age and sex were entered into a multivariable logistic regression model. The results demonstrated that IL‐6, lactate, and baseline SOFA score remained independently associated with organ dysfunction progression within 24 h (all *p* < 0.05). In contrast, the association between hsCRP and the outcome was attenuated after adjustment for confounding factors, and no longer reached statistical significance. Neither age nor sex showed an independent association with 24‐h organ dysfunction progression in the multivariable model (*p* > 0.05) (Table 3).

**TABLE 3:** Multivariable logistic regression analysis for 24‐h organ dysfunction progression.

Variable	Adjusted OR	95% CI	Statistical value	*p* value
Age (per 1‐year increase)	1.01	0.99–1.03	*Z* = 0.72	0.471
Male (yes vs. no)	1.15	0.63–2.08	*χ* ^2^ = 0.22	0.640
IL‐6 (ln‐transformed, per 1‐unit increase)	1.61	1.25–2.08	*Z* = 3.78	< 0.001
hsCRP (ln‐transformed, per 1‐unit increase)	1.18	0.92–1.52	*Z* = 1.33	0.183
Lactate (per 1 mmol/L increase)	1.41	1.14–1.75	*Z* = 3.14	0.002
Baseline SOFA score (per 1‐point increase)	1.14	1.05–1.24	*Z* = 3.32	0.001

### 3.4. Association Between IMCI and 24‐h Organ Dysfunction Progression

Univariable logistic regression analysis demonstrated that IMCI‐A was significantly associated with organ dysfunction progression within 24 h after ICU admission (odds ratio [OR] = 2.35, 95% CI: 1.78–3.11, *p* < 0.001). After adjustment for age, sex, and baseline SOFA score, IMCI‐A remained independently associated with 24‐h organ dysfunction progression (adjusted OR = 2.01, 95% CI: 1.48–2.73, *p* < 0.001).

To further address potential residual confounding, sensitivity analyses were performed by additionally incorporating clinically meaningful baseline and treatment‐related variables into the regression models. After further adjustment for WBC count, PLT count, and APACHE II score, the association between IMCI‐A and organ dysfunction progression remained significant (adjusted OR = 1.82, 95% CI: 1.31–2.53; *p* < 0.001). In the fully adjusted model that additionally included vasopressor dose, CRRT, mechanical ventilation, and glucocorticoid administration, IMCI‐A continued to show an independent association with 24‐h organ dysfunction progression (adjusted OR = 1.68, 95% CI: 1.18–2.39; *p* = 0.004).

**FIGURE 2:**
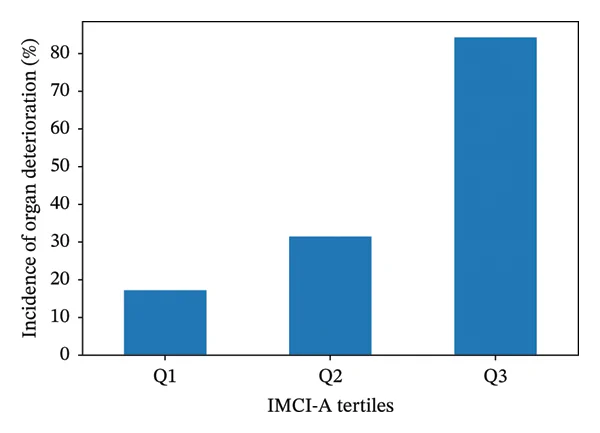
Incidence of 24‐h organ dysfunction progression across IMCI‐A tertiles.

In sensitivity analyses, the extended coupling index IMCI‐B was also independently associated with 24‐h organ dysfunction progression after adjustment for age, sex, and baseline SOFA score (adjusted OR = 1.89, 95% CI: 1.42–2.52, *p* < 0.001), and this association remained significant in the fully adjusted model (adjusted OR = 1.59, 95% CI: 1.12–2.25; *p* = 0.009), supporting the robustness of the observed association (Table 4). To address potential baseline SOFA‐related mathematical coupling and ceiling effect, sensitivity analyses were performed. IMCI‐A remained positively associated with 24‐h organ dysfunction progression across baseline SOFA tertiles, with adjusted ORs ranging from 1.42 to 1.67. After excluding patients in the highest baseline SOFA quartile, the association remained stable (adjusted OR = 1.72, 95% CI: 1.20–2.48; *p* = 0.003; AUC = 0.922), suggesting that the findings were not solely driven by baseline SOFA‐related mathematical coupling or ceiling effect (Supporting Table 2). For the assessment of baseline differences across IMCI‐A levels, patients were stratified into tertiles according to IMCI‐A. As shown in Table 5, age, sex distribution, and major comorbidities were generally comparable among the three groups. However, higher IMCI‐A tertiles were associated with greater inflammatory‐metabolic burden and more severe clinical status, including higher WBC count, IL‐6, hsCRP, lactate, baseline SOFA score, APACHE II score, and vasopressor requirements, as well as lower PLT count and higher proportions of CRRT and mechanical ventilation. These findings supported the use of multivariable and sensitivity analyses to account for baseline severity and treatment intensity. Furthermore, the incidence of 24‐h organ dysfunction progression increased stepwise across IMCI‐A tertiles (*p* for trend < 0.001), supporting the prognostic relevance of IMCI‐A beyond conventional baseline severity indicators (Figure 2).

**TABLE 4:** Association between IMCI and 24‐h organ dysfunction progression.

Index	Model	Adjustment variables	OR	95% CI	Statistical value	*p* value
IMCI‐A	Model 1	Unadjusted	2.35	1.78–3.11	*Z* = 5.62	< 0.001
IMCI‐A	Model 2	Age, sex, baseline SOFA score	2.01	1.48–2.73	*Z* = 4.21	< 0.001
IMCI‐A	Model 3	Model 2 + WBC count, PLT count, APACHE II score	1.82	1.31–2.53	*Z* = 3.56	< 0.001
IMCI‐A	Model 4	Model 3+vasopressor dose, CRRT, mechanical ventilation, glucocorticoid administration	1.68	1.18–2.39	*Z* = 2.89	0.004
IMCI‐B	Model 1	Unadjusted	2.18	1.67–2.86	*Z* = 5.04	< 0.001
IMCI‐B	Model 2	Age, sex, baseline SOFA score	1.89	1.42–2.52	*Z* = 4.32	< 0.001
IMCI‐B	Model 3	Model 2 + WBC count, PLT count, APACHE II score	1.72	1.26–2.35	*Z* = 3.43	0.001
IMCI‐B	Model 4	Model 3+vasopressor dose, CRRT, mechanical ventilation, glucocorticoid administration	1.59	1.12–2.25	*Z* = 2.60	0.009

*Note:* Model 1 was unadjusted. Model 2 was adjusted for age, sex, and baseline SOFA score. Model 3 was further adjusted for WBC count, PLT count, and APACHE II score. Model 4 was additionally adjusted for vasopressor dose, CRRT, mechanical ventilation, and glucocorticoid administration. IMCI‐A = ln(IL‐6) × ln(lactate).

**TABLE 5:** Baseline characteristics stratified by IMCI‐A tertiles.

Variable	Q1: Low IMCI‐A (*n* = 70)	Q2: Intermediate IMCI‐A (*n* = 70)	Q3: High IMCI‐A (*n* = 70)	Statistical value	*p* value
Age (year), mean ± SD	64.20 ± 12.80	65.10 ± 13.00	66.80 ± 12.50	*F* = 0.75	0.474
Male, *n* (%)	44 (62.86)	46 (65.71)	48 (68.57)	*χ* ^2^ = 0.51	0.776
Hypertension, *n* (%)	34 (48.57)	37 (52.86)	39 (55.71)	*χ* ^2^ = 0.73	0.696
Diabetes mellitus, *n* (%)	22 (31.43)	25 (35.71)	27 (38.57)	*χ* ^2^ = 0.79	0.673
Chronic kidney disease, *n* (%)	9 (12.86)	12 (17.14)	16 (22.86)	*χ* ^2^ = 2.43	0.297
Chronic cardiovascular disease, *n* (%)	18 (25.71)	21 (30.00)	24 (34.29)	*χ* ^2^ = 1.22	0.542
WBC count (× 10^9^/L), median (IQR)	10.80 (7.60–15.20)	13.60 (9.80–18.90)	17.40 (12.20–23.60)	*H* = 18.42	< 0.001
PLT count (× 10^9^/L), median (IQR)	142.00 (96.00–202.00)	116.00 (78.00–176.00)	86.00 (52.00–138.00)	*H* = 16.85	< 0.001
IL‐6 (pg/mL), median (IQR)	82.00 (55.00–130.00)	165.00 (105.00–260.00)	430.00 (260.00–720.00)	*H* = 82.60	< 0.001
hsCRP (mg/L), median (IQR)	68.00 (42.00–105.00)	96.00 (62.00–145.00)	142.00 (88.00–210.00)	*H* = 29.74	< 0.001
Lactate (mmol/L), mean ± SD	2.05 ± 0.58	3.02 ± 0.82	4.28 ± 1.32	*F* = 95.43	< 0.001
Baseline SOFA score, mean ± SD	8.20 ± 2.80	9.45 ± 3.05	10.96 ± 3.30	*F* = 14.31	< 0.001
APACHE II score, mean ± SD	20.40 ± 5.30	23.10 ± 5.80	26.20 ± 6.40	*F* = 17.23	< 0.001
Vasopressor dose (μg/kg/min), median (IQR)	0.16 (0.08–0.28)	0.25 (0.14–0.45)	0.42 (0.25–0.68)	*H* = 34.21	< 0.001
CRRT, *n* (%)	8 (11.43)	16 (22.86)	26 (37.14)	*χ* ^2^ = 12.81	0.002
Mechanical ventilation, *n* (%)	32 (45.71)	43 (61.43)	51 (72.86)	*χ* ^2^ = 10.83	0.004
Glucocorticoid administration, *n* (%)	22 (31.43)	27 (38.57)	31 (44.29)	*χ* ^2^ = 2.46	0.292

### 3.5. Predictive Performance of IMCI for 24‐h Organ Dysfunction Progression

To evaluate the predictive ability of IMCI for organ dysfunction progression within 24 h, ROC curves were constructed and the AUC was calculated, with comparisons made against IL‐6, lactate, and baseline SOFA score. IMCI‐A showed excellent discrimination (AUC = 0.929), with a higher AUC than IL‐6 (AUC = 0.777), lactate (AUC = 0.898), and baseline SOFA score (AUC = 0.839).

To compare the multiplicative and additive approaches, IMCI‐A was evaluated against IMCI‐additive. IMCI‐additive yielded an AUC of 0.914 (95% CI: 0.869–0.958), while IMCI‐A achieved a higher AUC of 0.929 (95% CI: 0.891–0.967). The difference was statistically significant by the DeLong test (*p* = 0.041), supporting the multiplicative formulation for capturing the coupled inflammatory–metabolic effect (Supporting Table 1).

**FIGURE 3:**
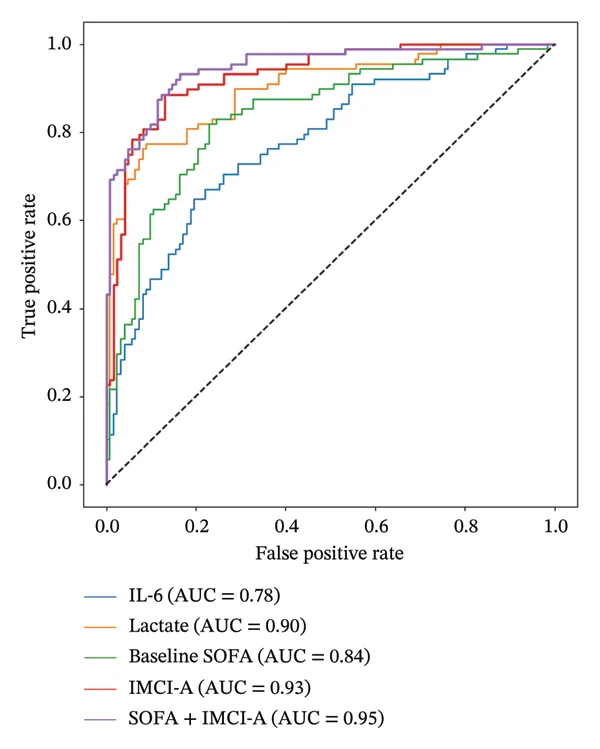
ROC curves of different indicators for predicting organ dysfunction progression within 24 h.

At the optimal cutoff value of 0.421, IMCI‐A achieved a sensitivity of 88.6%, specificity of 86.9%, accuracy of 87.6%, positive predictive value of 83.0%, and negative predictive value of 91.4%. When IMCI‐A was combined with baseline SOFA score, the AUC increased to 0.948. The VIF values for IMCI‐A and baseline SOFA score were both 1.46, indicating no severe multicollinearity. At the optimal cutoff value of 0.262, the combined model achieved a sensitivity of 93.2%, a specificity of 83.6%, and a negative predictive value of 94.4%, suggesting incremental predictive value beyond the baseline SOFA score (Figure 3).

To assess the robustness of predictive performance and the potential risk of overfitting, internal validation was performed using bootstrap resampling with 1000 repetitions. The original AUC of IMCI‐A was 0.929, with a bootstrap‐corrected mean AUC of 0.919 (95% CI: 0.890–0.961). For the combined SOFA + IMCI‐A model, the original AUC was 0.948, and the bootstrap‐corrected mean AUC was 0.939 (95% CI: 0.914–0.976). The discriminative performance after bootstrap validation was highly consistent with the original results, suggesting good stability and a low risk of overfitting.

### 3.6. Calibration Performance of IMCI for Prediction

**FIGURE 4:**
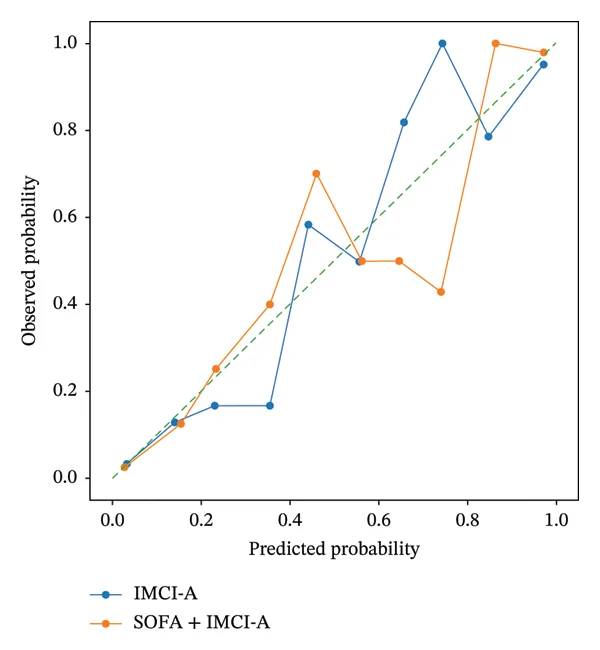
Calibration curves of IMCI‐A and the combined SOFA + IMCI‐A model for predicting organ dysfunction progression within 24 h. The dashed line represents the ideal reference line.

To evaluate the agreement between IMCI‐predicted probabilities and the observed incidence of organ dysfunction progression within 24 h, calibration curves were constructed and Brier scores were calculated. The results demonstrated good concordance between the predicted probabilities generated by IMCI‐A and the actual observed outcomes, with the overall distribution closely approximating the ideal reference line (45° line) and no evident systematic overestimation or underestimation (Figure 4). The Brier score for IMCI‐A was 0.101, indicating a low overall prediction error.

After incorporating IMCI‐A into the baseline SOFA score, calibration performance improved further. The calibration curve of the combined model more closely aligned with the ideal reference line, and the Brier score decreased to 0.086, suggesting that the integration of inflammation–metabolism coupling information with traditional organ function scoring provides superior overall predictive accuracy and stability.

### 3.7. Decision Curve Analysis Results for IMCI‐Based Prediction

**FIGURE 5:**
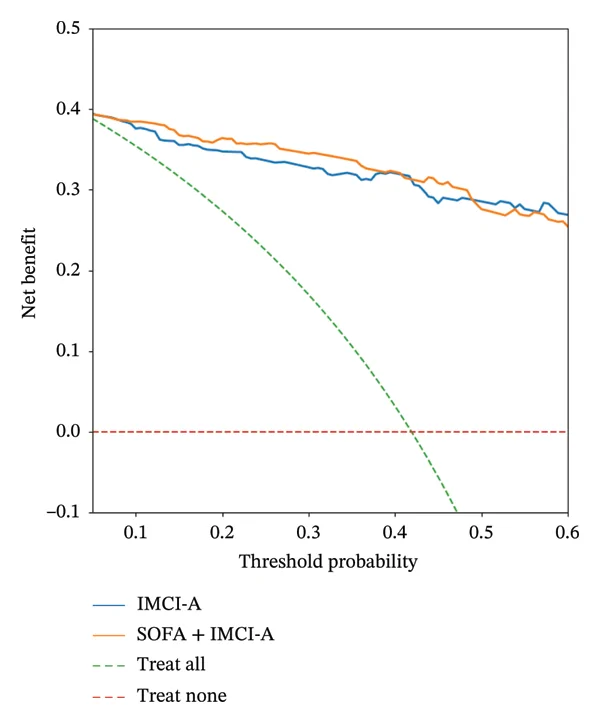
Decision curve analysis curves of IMCI‐A and the combined SOFA + IMCI‐A model for predicting organ dysfunction progression within 24 h. The *x*‐axis represents threshold probability, and the *y*‐axis represents net benefit. The dashed lines indicate the “treat all” and “treat none” strategies.

To assess the potential clinical utility of IMCI in decision‐making, decision curve analysis was performed. The results showed that across a wide range of threshold probabilities (0.05–0.60), both IMCI‐A and the combined SOFA + IMCI‐A model achieved higher net benefits compared with the “treat all” and “treat none” strategies (Figure 5). Notably, the SOFA + IMCI‐A model demonstrated slightly higher or comparable net benefit to IMCI‐A alone across most threshold probability intervals, suggesting that this combined approach may provide greater potential benefit for patient management in clinical risk stratification and intervention decision‐making.

## 4. Discussion

In this retrospective cohort of septic shock patients, we developed and internally validated a novel IMCI for early prediction of organ dysfunction progression within 24 h after ICU admission. IL‐6 and blood lactate were independently associated with early SOFA progression, and their integration into IMCI significantly improved risk stratification, with IMCI‐A outperforming individual biomarkers and baseline SOFA. Notably, combining IMCI with SOFA further enhanced discrimination, calibration, and clinical net benefit, underscoring the prognostic value of inflammation–metabolism coupling in septic shock.

Previous studies have extensively evaluated individual inflammatory or metabolic biomarkers for outcome prediction in sepsis. IL‐6 has consistently been identified as a robust indicator of systemic inflammatory activation and disease severity, with reported AUC values generally ranging from 0.70 to 0.80 for mortality or organ dysfunction prediction [14]. Similarly, lactate remains a cornerstone marker reflecting impaired tissue perfusion and cellular metabolic stress, and its prognostic value in septic shock has been repeatedly validated [15, 16]. However, despite their clinical utility, these single markers capture only isolated aspects of the complex sepsis pathophysiology. Our findings are concordant with prior reports demonstrating independent associations of IL‐6 and lactate with adverse outcomes but extend the literature by showing that their interaction conveys substantially greater predictive information than either marker alone.

The superiority of IMCI over single indicators is biologically plausible. Sepsis is characterized by a tightly intertwined inflammatory and metabolic response, in which cytokine‐driven immune activation promotes mitochondrial dysfunction, impaired oxidative phosphorylation, and dysregulated glycolysis, ultimately leading to lactate accumulation [17, 18]. Conversely, metabolic stress and tissue hypoxia further amplify inflammatory signaling through hypoxia‐inducible and stress‐response pathways [19]. In this study, by mathematically coupling IL‐6 and lactate on a logarithmic scale, IMCI integrates inflammatory and metabolic information into a unified index. This formulation was further supported by the comparison with a simple additive model, in which IMCI‐A showed higher discriminative performance by the DeLong test. These results suggest that the multiplicative formulation may better capture the amplified risk associated with simultaneous elevation of inflammatory and metabolic markers. This interaction‐based framework represents an alternative to conventional additive or multivariable models and may contribute to its improved discrimination for early organ deterioration.

Compared with established clinical scores, such as SOFA, IMCI offers complementary rather than redundant information. SOFA reflects the extent of organ dysfunction at a given time point [13], but does not directly capture upstream inflammatory activation or metabolic stress. In our study, IMCI‐A remained associated with 24‐h organ dysfunction progression after adjustment for baseline SOFA score and other clinically relevant variables, suggesting that inflammation–metabolism coupling may capture early pathophysiological changes not fully captured by conventional scoring. However, defining the outcome as ΔSOFA while also adjusting for baseline SOFA introduces potential structural coupling, because baseline SOFA is both incorporated into the change score and included as a model covariate. Moreover, patients with high baseline SOFA scores have less opportunity for further score increases and may therefore be less likely to reach the ΔSOFA ≥ 2 threshold because of ceiling effects. To assess the robustness of our findings, we performed analyses stratified by baseline SOFA tertiles and repeated the analysis after excluding patients in the highest baseline SOFA quartile. The association remained consistent, suggesting that it was unlikely to be entirely driven by baseline SOFA distribution. Nevertheless, these analyses cannot fully eliminate structural coupling or ceiling effects, and the findings should therefore be interpreted as an association with early SOFA progression rather than definitive evidence of prediction independent of baseline score‐related bias. The combined SOFA + IMCI model achieved the highest AUC, best calibration, and greatest net clinical benefit, supporting the potential value of integrating mechanistic biomarkers with clinical scoring systems. Although higher IMCI‐A tertiles were associated with greater inflammatory‐metabolic burden and clinical severity, IMCI‐A remained independently associated with 24‐h organ dysfunction progression after adjustment for baseline severity and treatment‐related variables. These findings suggest that IMCI‐A is not merely a surrogate for disease severity and may provide additional prognostic information.

Notably, hsCRP lost statistical significance after multivariable adjustment, which aligns with prior literature indicating that CRP is a relatively nonspecific and temporally delayed inflammatory marker in acute sepsis [20]. In contrast, IL‐6 rises rapidly and more directly reflects cytokine‐driven immune activation, reinforcing its suitability for early prognostic modeling [21]. Sensitivity analyses using the extended index IMCI‐B, which incorporated both IL‐6 and hsCRP, confirmed the robustness of the coupling concept, while highlighting IL‐6 as the dominant inflammatory component in early septic shock.

To further account for baseline disease severity and treatment intensity, we incorporated WBC count, PLT count, APACHE II score, vasopressor dose, CRRT, mechanical ventilation, and glucocorticoid administration into the sensitivity analyses. The association between IMCI‐A and 24‐h organ dysfunction progression remained stable after these adjustments, suggesting that its predictive value was not solely explained by baseline severity or treatment‐related confounding. Nevertheless, residual confounding cannot be completely excluded because of the retrospective study design.

From a clinical perspective, IMCI‐A is based on routinely available early laboratory data and may support more individualized early management in septic shock. Patients with low IMCI‐A levels may represent a relatively low‐risk subgroup, in whom unnecessary escalation of monitoring or organ support could potentially be avoided when clinical status is stable. Conversely, patients with high IMCI‐A levels may require closer reassessment of SOFA components, lactate dynamics, hemodynamic status, urine output, and respiratory function, as well as earlier consideration of intensified monitoring or intervention. Therefore, IMCI‐A should be regarded as an adjunct to clinical judgment rather than a stand‐alone decision‐making tool.

However, this study has several limitations. First, its single‐center retrospective design and lack of external validation limit the generalizability of the findings. The observed model performance may partly reflect center‐specific patient characteristics, clinical practices, and laboratory procedures; therefore, favorable internal validation should not be interpreted as evidence of broad clinical applicability. Residual confounding also remains possible because unmeasured factors, including infection source, antimicrobial timing, fluid resuscitation, and changes in organ support, may have influenced the results. In addition, patients with missing key data were excluded from the complete‐case analysis. Because the missing‐data mechanism could not be definitively determined, some missingness may have been clinically driven, such as early death or rapid deterioration before completion of laboratory testing or 24‐h SOFA assessment, potentially introducing selection bias. Moreover, defining the outcome as ΔSOFA ≥ 2 while adjusting for baseline SOFA may introduce structural coupling, whereas high baseline SOFA scores may restrict further increases and produce ceiling effects. Although the associations remained consistent after stratification by baseline SOFA and exclusion of patients in the highest baseline SOFA quartile, these analyses cannot fully eliminate such bias. Future studies should consider baseline‐adjusted 24‐h SOFA, longitudinal SOFA trajectories, or organ‐specific changes. Second, only measurements obtained within the first 24 h were analyzed, whereas serial biomarker changes may provide additional prognostic information. Third, prospective multicenter studies with independent external validation are required before clinical implementation. Finally, incorporating additional inflammatory and metabolic markers may further refine the coupling framework.

## 5. Conclusion

Coupling inflammatory intensity with metabolic dysregulation provides a powerful and clinically meaningful approach for early prediction of organ dysfunction progression in septic shock. IMCI complements traditional organ function scores and may facilitate earlier, more precise risk stratification. Prospective multicenter studies are needed to validate these findings and to explore whether IMCI‐guided management strategies can translate into improved clinical outcomes.

**Nomenclature**
ICU: Intensive care unit
SOFA: Sequential organ failure assessment
ΔSOFA: Change in SOFA score
IMCI: Inflammation–metabolism coupling index
IMCI‐A: Primary inflammation–metabolism coupling index(ln[IL‐6] × ln[lactate])
IMCI‐B: Extended inflammation–metabolism coupling index ([Z(ln(IL‐6)) + Z(ln(hsCRP))] × Z(ln(lactate))
IL‐6: Interleukin‐6
hsCRP: High‐sensitivity C‐reactive protein
ROC: Receiver operating characteristic
AUC: Area under the curve
OR: Odds ratio
CI: Confidence interval
ELISA: Enzyme‐linked immunosorbent assay

## Author Contributions

Weiying Ding: data curation, resources, methodology, software, validation, and writing–original draft. Qiang She: formal analysis, investigation, project administration, and writing–original draft. Yingjie Geng: conceptualization, supervision, visualization, and writing–review and editing.

## Funding

No funding was received for this manuscript.

## Ethics Statement

The study protocol was approved by the Beijing Shijingshan Hospital Ethics Committee (No. SJS2026‐LW106) and conducted in accordance with the principles of the Declaration of Helsinki.

## Consent

Owing to the retrospective nature of the study, the requirement for informed consent was waived.

## Conflicts of Interest

The authors declare no conflicts of interest.

## Supporting Information

Additional supporting information can be found online in the Supporting Information section.

## Supporting information


**Supporting Information** The supporting information for this article includes Supporting Table 1, which compares the predictive performance of the additive and multiplicative inflammation–metabolism models, and Supporting Table 2, which presents sensitivity analyses addressing potential baseline SOFA‐related mathematical coupling and ceiling effects.

## Data Availability

The data that support the findings of this study are available from the corresponding author upon reasonable request.
