# Supplementary material for: A Coupled Inflammation–Metabolism Index for Early Prediction of Organ Dysfunction Progression Within 24 h After ICU Admission in Septic Shock
Source: Emerg Med Int. 2026 Sep 9;2026:1308518. doi: 10.1155/emmi/1308518 (PMC13559166; doi:10.1155/emmi/1308518)
Supplement: Supplementary file 1 [file EMMI-2026-1308518-s001.docx]

**Supplementary Table 1. Comparison between additive and multiplicative inflammation-metabolism models**

| Model | Formula | AUC | 95% CI | DeLong P value |
| --- | --- | --- | --- | --- |
| Additive model | Z[ln(IL-6)]+Z[ln(lactate)] | 0.914 | 0.869-0.958 | — |
| Multiplicative model, IMCI-A | ln(IL-6)×ln(lactate) | 0.929 | 0.891-0.967 | 0.041 |

The DeLong P value indicates the comparison of AUC between IMCI-A and IMCI-additive.

**Supplementary Table 2. Sensitivity analyses addressing baseline SOFA-related mathematical coupling and ceiling effect**

| Analysis | n | Events, n (%) | Adjusted OR of IMCI-A | 95% CI | P value | AUC |
| --- | --- | --- | --- | --- | --- | --- |
| Overall cohort | 210 | 88 (41.9) | 1.68 | 1.18-2.39 | 0.004 | 0.929 |
| Low baseline SOFA tertile | 70 | 25 (35.7) | 1.55 | 1.08-2.24 | 0.018 | 0.887 |
| Intermediate baseline SOFA tertile | 70 | 33 (47.1) | 1.67 | 1.15-2.42 | 0.007 | 0.903 |
| High baseline SOFA tertile | 70 | 30 (42.9) | 1.42 | 1.01-2.01 | 0.045 | 0.876 |
| Excluding highest baseline SOFA quartile | 158 | 65 (41.1) | 1.72 | 1.20-2.48 | 0.003 | 0.922 |
